# Effects of elevated temperature and CO_2_ on aboveground-belowground systems: a case study with plants, their mutualistic bacteria and root/shoot herbivores

**DOI:** 10.3389/fpls.2013.00445

**Published:** 2013-11-11

**Authors:** James M. W. Ryalls, Markus Riegler, Ben D. Moore, Goran Lopaticki, Scott N. Johnson

**Affiliations:** Hawkesbury Institute for the Environment, University of Western SydneyRichmond, NSW, Australia

**Keywords:** alfalfa, aphid, lucerne, *Medicago sativa*, nodule, root, weevil

## Abstract

Interactions between above- and belowground herbivores have been prominent in the field of aboveground-belowground ecology from the outset, although little is known about how climate change affects these organisms when they share the same plant. Additionally, the interactive effects of multiple factors associated with climate change such as elevated temperature (eT) and elevated atmospheric carbon dioxide (eCO_2_) are untested. We investigated how eT and eCO_2_ affected larval development of the lucerne weevil (*Sitona discoideus*) and colonization by the pea aphid (*Acyrthosiphon pisum*), on three cultivars of a common host plant, lucerne (*Medicago sativa*). *Sitona discoideus* larvae feed on root nodules housing N_2_-fixing rhizobial bacteria, allowing us to test the effects of eT and eCO_2_ across trophic levels. Moreover, we assessed the influence of these factors on plant growth. eT increased plant growth rate initially (6, 8 and 10 weeks after sowing), with cultivar “Sequel” achieving the greatest height. Inoculation with aphids, however, reduced plant growth at week 14. eT severely reduced root nodulation by 43%, whereas eCO_2_ promoted nodulation by 56%, but only at ambient temperatures. Weevil presence increased net root biomass and nodulation, by 31 and 45%, respectively, showing an overcompensatory plant growth response. Effects of eT and eCO_2_ on root nodulation were mirrored by weevil larval development; eT and eCO_2_ reduced and increased larval development, respectively. Contrary to expectations, aphid colonization was unaffected by eT or eCO_2_, but there was a near-significant 10% reduction in colonization rates on plants with weevils present belowground. The contrasting effects of eT and eCO_2_ on weevils potentially occurred through changes in root nodulation patterns.

## INTRODUCTION

Many studies report on plant-mediated interactions between spatially separated insect herbivores that live above- and belowground, yet few studies have considered these interactions in the context of global climate change. Climate change involves multiple factors such as warming and rising atmospheric carbon dioxide (CO_2_) concentrations. While the effects of predicted increases in global average surface temperatures (by 1–4°C within this century) and atmospheric CO_2_ concentrations (from current levels of 400 to over 550 μmol mol^-1^ by 2050) on insect-plant interactions have been characterized separately, only a handful of studies have considered more than one climate change variable simultaneously (Robinson et al., 2012; Stevnbak et al., 2012; Murray et al., 2013). Moreover, the role of plant microbes, such as mutualistic rhizobial bacteria which form intimate associations with plants, have not yet been investigated. Combining trophic complexity and multiple climatic factors is challenging but necessary to provide a more holistic insight into the mechanisms underpinning insect–plant interactions (Ryalls et al., 2013).

All legumes form symbioses with rhizobial bacteria that fix atmospheric nitrogen (N_2_) and are carried in root nodules (Haag et al., 2013). Root nodulation is important for the larval development of *Sitona* weevil species (Coleoptera: Curculionidae), including *S. discoideus*, which feeds on lucerne root nodules throughout its larval growth stages (Allen, 1971; Goldson et al., 1988a; Vink and Phillips, 2007).* Sitona discoideus* therefore has the potential to reduce N-fixation in lucerne by damaging root nodules (Keane and Barlow, 2002). The net effect of *S. discoideus* on nodule numbers depends on the ability of the plant to compensate for nodule loss (Quinn and Hall, 1992). The abundance and size of root nodules can also be influenced by climate change. For example, it is widely reported that root nodulation (and biological N-fixation) increases in response to elevated CO_2_ concentrations (eCO_2_; Ryle and Powell, 1992; Lüscher et al., 2000) but decreases with elevated temperatures (eT; Munns et al., 1979; Zahran, 1999).

Root-feeding organisms can influence the chemical composition and biomass of aboveground plant parts, which, in turn, can influence the survival of aboveground insect herbivores (Soler et al., 2012). A recent meta-analysis (Johnson et al., 2012) confirmed that root herbivory by beetle larvae usually had beneficial effects on aboveground aphids. This potentially arises through impaired root function and stress-related accumulation of N in the foliage (Masters et al., 1993). Given that, unlike in most other plants, rhizobial nodules underpin N balance in legumes, there is good reason to hypothesize that the presence of belowground herbivores that specifically target root nodules (and therefore have a greater impact on N uptake than generalized root feeders) will reverse this trend for better aphid performance on plants with root herbivores.

The net effect on plant growth when subjected to above- and belowground herbivory under the influence of multiple climatic factors is unknown and untested. Here we aim to characterize this in a model legume system that incorporates multiple organisms, including *Rhizobium* bacteria, the nodule-feeding lucerne weevil (*S. discoideus*) and the sap-sucking pea aphid (*Acyrthosiphon pisum;* Hemiptera: Aphididae), with both insects feeding on a common host plant, lucerne (*Medicago sativa* L.; Fabales: Fabaceae). Lucerne is the most important and widely grown temperate forage legume globally (Small, 2011; Bouton, 2012). *Acyrthosiphon pisum* is a widespread pest of lucerne and a concerted program of incorporating aphid resistance into cultivars since the introduction of lucerne-feeding aphids, including *A. pisum*, to Australia in 1980, has helped to control aphid populations. Occasional outbreaks, however, driven by environmental factors including climatic variability and interactions with other trophic groups (e.g., release from natural enemies), still occur (Zarrabi et al., 1995; Humphries et al., 2012). Moreover, the susceptibility of different cultivars to *A. pisum* can be modified by such factors (Ryalls et al., 2013), and we therefore included cultivars with moderate (“Trifecta”), low (“Sequel”) and no (“Hunter River”) *A. pisum* resistance.

We present a novel case study that examines the interactive effects of temperature (daytime temperature 26 and 30°C, aT and eT, respectively) and atmospheric CO_2_ concentration (400 and 640 ppm, aCO_2_ and eCO_2_, respectively) on the interactions between lucerne cultivars, rhizobial bacteria, a root herbivore and an aphid. Specifically, we set out to investigate how: (i) aphid herbivory affects plant growth (height) under eCO_2_ and eT; (ii) eCO_2_ and eT affect root nodulation and the performance of *S. discoideus*; (iii) the presence of nodule-feeding *S. discoideus* affects the ability of *A. pisum* to successfully colonize and reproduce on three cultivars of lucerne of varying resistance to *A. pisum* under eCO_2_ and eT. We hypothesized that: (i) eCO_2_ and eT would promote plant growth but aphid herbivory would reduce the rate of growth; (ii) eCO_2_ and eT would promote and reduce nodulation, respectively, and *S. discoideus* would perform better (i.e., complete larval development) at lower temperatures and higher CO_2_ concentrations; and finally, (iii) nodule-feeding by *S. discoideus* would have a negative impact on aphid abundance via impaired root function and decreased plant quality. For the purpose of this case study, weevil emergence refers to the number of plants with *S. discoideus* that grew to adulthood and emerged from the soil.

## MATERIALS AND METHODS

### GROWTH CONDITIONS AND EXPERIMENTAL DESIGN

Four glasshouse chambers, providing two atmospheric CO_2_ concentrations (400 and 640 μmol mol^-1^, aCO_2_ and eCO_2_ respectively) and two temperature treatments (daytime temperature 26 and 30°C, aT and eT, respectively) combined factorially, were used in this study. aT (maintained at 26/18°C day/night on a 15L:9D cycle) represents the daily average temperature data (November to May) for Richmond, NSW (latitude -33.611098, longitude 150.742368; Australian Bureau of Meteorology) and eT (30/22°C day/night) was based on the predicted maximum temperature increase for this region within this century (CSIRO, Bureau of Meteorology, 2007). Humidity was controlled at 55%. Photosynthetic active radiation (PAR) was measured every hour between 10 am and 3 pm. PAR ranged from 210 to 580 μmol m^-2^ s^-1^; no significant differences in PAR were observed between chambers overall. As an attempt to compensate for the effects of pseudo replication (though not excluding them) arising from the lack of replication of climates (climate-temperature combinations), we rotated the position of pots randomly within chambers twice weekly (Quirk et al., 2013; Sherwin et al., 2013). The environmental conditions within the chambers were logged and monitored continuously throughout the experiment to maintain temperature and CO_2_ differences between chambers and temperature readings were cross-checked with transportable temperature loggers, as in Murray et al. (2013).

Lucerne seeds (sourced from Seedmark, Adelaide, South Australia) were inoculated with *Rhizobium* bacteria one hour prior to planting by submerging in a solution containing 250 g Nodule N lucerne seed inoculant (New Edge Microbials, Albury, NSW, Australia) and 800 mL distilled water. In each chamber, 120 lucerne seeds (40 of each of the three cultivars) were individually planted in 70 mm pots filled with sieved (2 mm) local loamy-sand soil collected from the Hawkesbury Forest Experiment in Richmond, NSW (Barton et al., 2010). Soil was kept moist by watering daily (c. 15 mL). *Acyrthosiphon pisum* cultures, reared from a single parthenogenetic adult female collected in Richmond, NSW were maintained at 22/14°C day/night on the susceptible cultivar “Hunter River” until required. Additionally, 20 sexually mature *S. discoideus* adults, also collected by sweep-netting in July 2012 from local lucerne fields in Richmond, NSW, were reared on “Hunter River” and eggs were collected every 24 h and stored on damp filter paper at 4°C until required. Hatching success was assessed and confirmed (>95% hatched within 5 days) by placing 200 eggs on 10 petri dishes at 25°C. “Hunter River” was included as an experimental cultivar to provide a baseline for comparison with the resistant cultivars.

### EXPERIMENTAL PROCEDURE

Plants in each of the four chambers (aCO_2_ × aT; eCO_2_ × aT; aCO_2_ × eT; eCO_2_ × eT) were distributed randomly among four treatments: weevils only (W), aphids only (A), weevils and aphids (WA) and no insects or control (C), giving 10 replicates of each treatment per cultivar in each chamber. When plants were six weeks old, half of the plants (treatments W and WA) were inoculated with 20 *S. discoideus* eggs per plant. This egg density (6027 eggs per m^2^) resembles *S. discoideus* eggs densities recorded in NSW during June (5185 eggs per m^2^; Aeschlimann, 1983). Eggs were placed on top of the soil beside the stem of each plant. After a further 4 weeks, two teneral adult pea aphids were transferred to each plant in treatments A and WA. Plants were placed on plastic plinths within water-filled trays, which acted as moats to prevent aphid movement between plants. Aphid presence was recorded 7 and 14 days later and aphids were removed after 21 days. Six weeks after egg inoculation, plants were checked every 12 h for emerging adult weevils (i.e., those that had completed larval development and emerged from the soil leaving an exit hole). One week after aphids were removed, roots were separated from the soil and the numbers of root nodules were counted. Plant heights (from ground level to the base of the highest leaf) were measured 6 (weevil egg inoculation period), 8, 10 (aphid inoculation period) and 14 (harvest period) weeks after planting. Lucerne height is strongly correlated with biomass (Michalk and Herbert, 1977) so this represents an excellent non-destructive proxy for plant growth.

### STATISTICAL ANALYSES

#### Plant responses

The effects of CO_2_ and temperature on lucerne height measurements over 8 weeks were determined using general linear models within the R statistical interface v2.15.1. The main effect terms of “CO_2_,” “temperature,” “cultivar” and “treatment” (i.e., aphid- and weevil-treated), as well as their associated interactions, were included. The effects of temperature, CO_2_, cultivar and weevil presence on the number of nodules were assessed using multifactorial ANOVA in R. The dependent variable “nodule number” was log-transformed to standardize residuals. Pairs of mean estimated effects were compared using a Tukey-Kramer *post hoc* test. ANOVA was used to compare mean differences in root mass between weevil-treated and untreated plants. Root mass was square root transformed to normalise data.

#### Insect responses

The effects of temperature and CO_2_ treatment on the proportion of plants containing weevils that reached adulthood and emerged from the soil were assessed using a generalized linear model with a binomial error structure and logit link function within R. The full model included main effect terms for “cultivar,” “temperature,” “CO_2_” and “aphid-treatment” (i.e., whether plants had aphids applied), as well as the interactions between these terms. The effects of CO_2_, temperature, cultivar and weevil presence on aphid success were determined using a generalized linear model with a binomial error structure and logit link function, using aphid success (colonization) as the dependent variable. Models were reduced in a stepwise manner by removing the non-significant terms in order of least significance and plants that had died before harvest were not included in analyses.

## RESULTS

### PLANT RESPONSES

#### Height

**FIGURE 1:**
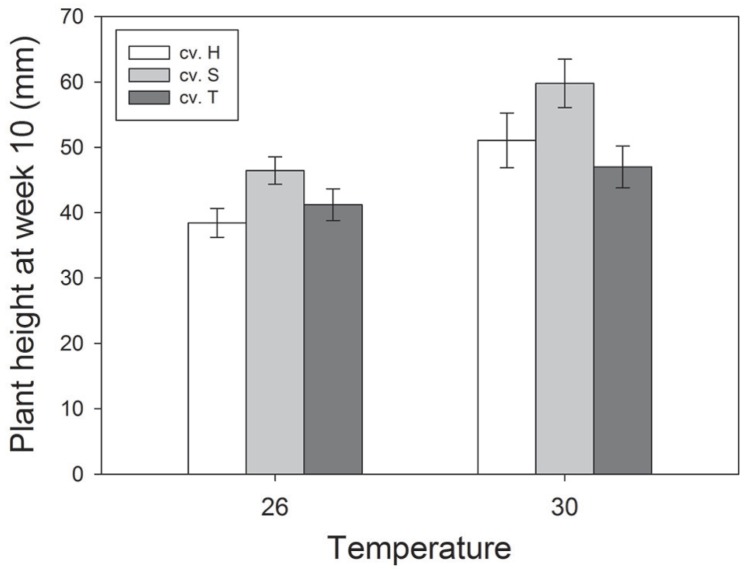
**Effects of temperature and cultivar (Hunter River, H; Sequel, S and Trifecta, T) on plant growth (height) 10 weeks after sowing.** Mean values (± standard errors) of significant treatment factors (temperature and cultivar) in the final model shown.

**FIGURE 2:**
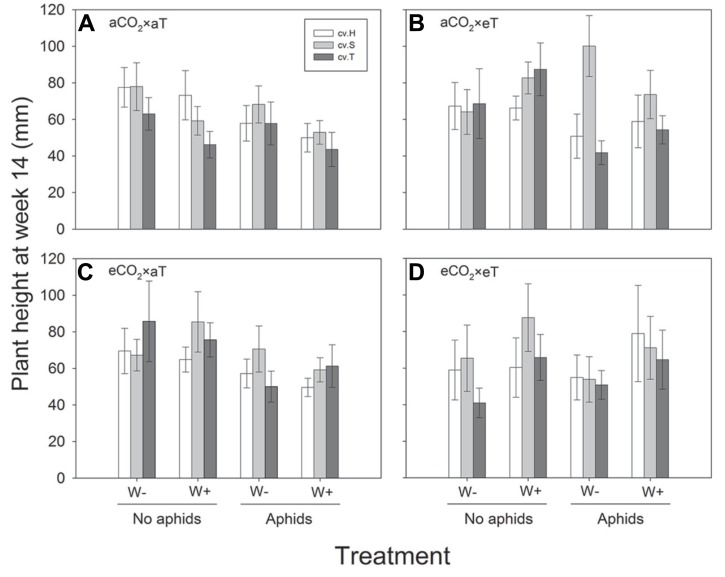
**Height of three lucerne cultivars (Hunter River, H; Sequel, S and Trifecta, T) 14 weeks after sowing in treatments with and without aphids and weevils (plants with weevils, W+; plants without weevils, W-).** Graphs **(A–D)** represent the four “CO_2_ × temperature” treatments. Mean values (± standard errors) of significant treatment factors (cultivar, aphid presence, CO_2_: temperature and temperature: weevil presence) in the final model shown.

Plant height was significantly greater at higher temperatures at week 6 (*F*_1,468_ = 59.03; *P* < 0.001), week 8 (*F*_1,468_ = 40.11; *P* < 0.001) and week 10 (*F*_1,468_ = 8.56; *P* = 0.004), whereas CO_2_ did not affect plant height in these weeks (see **Figure 1** for significant effects at week 10). In addition, there were significant differences in height between cultivars in these same weeks (*F*_2,468_ = 14.52, *P* < 0.001; *F*_2,468_ = 9.03; *P* < 0.001; *F*_2,468_ = 9.45;* P* < 0.001), with Sequel being significantly bigger than the other two cultivars overall (**Table 1**). At week 14, aphids reduced plant height (*t*_1,195_ = -2.637; *P* = 0.008) and there was a significant interaction between CO_2_ and temperature (*F*_2,468_ = 8.21; *P* = 0.004) whereby eCO_2_ increased plant growth at 26°C (from 57.5 ± 4.2 mm at aCO_2_ to 66.4 ± 4.2 mm at eCO_2_) but not at 30°C. The presence of weevils also interacted with temperature effects (*F*_2,468_ = 6.94; *P* = 0.009), with weevils decreasing overall plant height at 26°C (from 66.9 ± 4.4 mm without weevils to 58.1 ± 3.9 mm with weevils) but not at 30°C (see **Figure 2** for all significant treatment factors).

**Table 1:** Effect of cultivar on plant height, root nodulation, aphid colonization and weevil emergence.

Cultivar	Plant height (mm)	Number of nodules per plant	Plants colonized by aphids (%)	Weevil emergence (%)
Hunter River	46.3 ± 2.1	9.8 ± 0.8	27.5	6.3
Sequel	54.8 ± 2.1	12.4 ± 1.1	20.0	13.8
Trifecta	45.6 ± 1.9	7.5 ± 0.9	32.5	8.8
Test results	*F*_2,468_ = 3.96; *P* = 0.020*	*F*_2,409_ = 9.585; *P* < 0.001*	χ^2^(*df* = 2) 3.324; *P *= 0.190	χ^2^(*df* = 2) 2.899; *P* = 0.235

*Overall means (± standard error) and frequencies shown.*

* Significance indicated by linear model outputs and ANOVA (χ^2^) test results.

#### Nodulation and root mass

**FIGURE 3:**
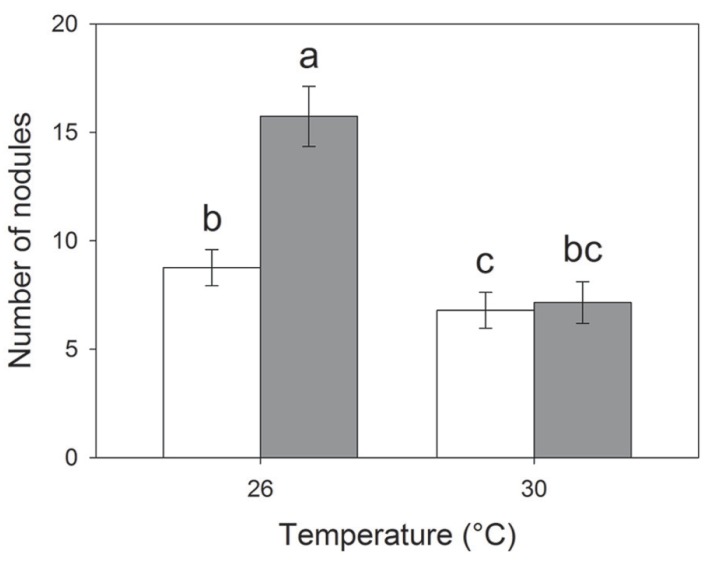
**Effects of temperature on the number of root nodules per plant under eCO_2_** (closed bars) and aCO_**2**_ (open bars). Mean values (± standard errors) shown. Bars with the same letters were not significantly different (*P* < 0.05).

Elevated temperature resulted in reduced nodulation (*F*_1,409_ = 34.78; *P* < 0.001) but the interaction between CO_2_ and temperature also affected root nodulation (*F*_1,417_ = 4.11; *P* = 0.042); nodulation increased under eCO_2_ at 26°C (Tukey HSD; *P* < 0.01) but not at 30°C (Tukey HSD; *P* = 0.983; **Figure 3**). Plants with *S. discoideus* had a significantly higher number of nodules and greater root mass than those without (**Table 2**).

**Table 2:** Mean (± standard error) numbers of nodules and root mass between weevil-treated and -untreated plants.

	*N*	Number of nodules	Root mass (g)
Weevil-treated	209	11.71 ± 0.94	0.093 ± 0.004
Weevil-untreated	209	8.08 ± 0.57	0.071 ± 0.003
Test results		*F*_1,417_ = 11.52;	*F*_1,417_ = 11.55;
		*P* < 0.001	*P*< 0.001

*Summary statistical results of tests for differences between the variable means are provided.*

### INSECT RESPONSES

#### Weevils

**FIGURE 4:**
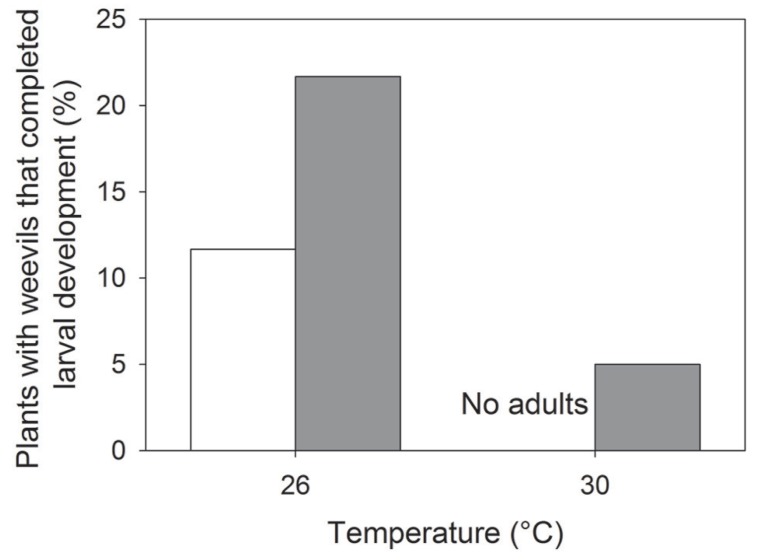
**Effects of temperature on the proportion of plants containing weevils that reached adulthood and emerged from the soil at aCO_2_** (open bars) and eCO_**2**_ (closed bars). All columns were significantly different (*P* < 0.01) from one another.

Elevated temperature had a negative effect on weevil emergence (i.e., the proportion of plants with emerging weevils; *z*_1,23__4_ = -3.29; *P* < 0.001), whereas eCO_2_ positively affected weevil emergence (*z*_1,23__4_ = 2.00; *P* = 0.045; **Figure 4**). Weevil emergence was not influenced by cultivar type (**Table 1**). These responses resembled those seen for nodulation, i.e., negative and positive effects of eT and eCO_2_, respectively.

#### Aphids

*Acyrthosiphon pisum* was not significantly affected by changes in temperature and CO_2_ or the presence of weevils, although there was a trend for the proportion of plants that were successfully colonized by *A. pisum* to be lower in plants inoculated with *S. discoideus* eggs [χ^2^(*df* = 1) 3.15; *P* = 0.076].

## DISCUSSION

Our study is first to incorporate the effects of both eT and eCO_2_ on a system combining above- and belowground insect herbivores. The results demonstrate that eT can negate the positive effects of eCO_2_ on lucerne nodulation and herbivory belowground, an important consideration for determining future outcomes of climate change.

Elevated temperature promoted plant growth initially and aphid herbivory reduced plant growth at week 14. The reduction in height of plants with aphids present, however, was facilitated by root feeding by *S. discoideus*, indicated by an interaction between temperature and weevil presence at week 14; the presence of *S. discoideus* decreased lucerne height at aT but not at eT. Few weevils, however, emerged at eT, suggesting that they did not account for the changes in plant growth observed at eT. 30°C may represent an upper developmental threshold (i.e., the temperature at which development rate becomes suboptimal) for *S. discoideus*. Arbab et al. (2008) identified 28°C as the optimum temperature for *S. discoideus* development with development time decreasing with increasing temperatures within the range of 8.5 to 28°C (from a hatching rate of 69.01 ± 0.92 days to 8.46 ± 0.14 days, respectively). Additionally, egg development rates at 26°C matched rates at 30°C and they calculated the upper developmental temperature threshold as 30 to 32.6°C. Temperature increases toward the thermal optimum may positively influence the abundance of *S. discoideus*, whereas temperatures above 30°C may negatively affect *S. discoideus* numbers.

Effects of CO_2_ on root nodulation mirrored their effects on the emergence of *S. discoideus* at 26°C, suggesting that *S. discoideus* performance was influenced by the number of root nodules on lucerne. eCO_2_ has been shown to promote nodulation and performance of another *Sitona* species (*S. lepidus*) on white clover (*Trifolium repens*) (Johnson and McNicol, 2010). In that study, the effects of temperature were not tested, though the authors speculated that enhanced nodulation and *S. lepidus* larval performance seen under eCO_2_ might be tempered by eT. In the present study, we observed that eT negated the positive effects of eCO_2_ on nodulation. Such negation of eCO_2_ effects by eT has also been found in aboveground insect–plant interactions (Murray et al., 2013) and further emphasizes the need to consider multiple climate change factors simultaneously when assessing the effects of global environmental change.

Modest root herbivory often produces compensatory responses by the plant which may increase root growth to offset losses due to herbivory (Andersen, 1987; Brown and Gange, 1990; Blossey and Hunt-Joshi, 2003). In the present study, we observed even larger increases in root growth and nodulation in the presence of *S. discoideus* above and beyond those observed in plants without *S. discoideus*, i.e., overcompensation. Overcompensatory nodulation has also been demonstrated in lucerne in response to *Sitona hispidulus*, which, after 10 days of nodule-feeding, increased the number of nodule units per plant by 89 ± 9% compared to 25 ± 7% in control plants (Quinn and Hall, 1992). Our study used egg densities (c. 6000 eggs per m^2^) similar to those seen in the field in NSW, Australia during June (c. 5000 eggs per m^2^), see Section “Experimental Procedure” (Aeschlimann, 1983). Higher egg densities, such as those reported by Aeschlimann and Vitou (1988) of 9500 eggs per m^2^, however, could result in root herbivory that was too severe for compensatory growth to occur.

Few changes were observed in aphids feeding aboveground, although numbers of aphids that colonized plants (i.e., remained on the plant and reproduced) were low, making aboveground-belowground and cultivar interactions difficult to interpret. Johnson et al. (2012) confirmed that, generally, aboveground aphids are positively affected by belowground root feeders. Unlike most other root herbivores, *S. discoideus* attacks root nodules (and therefore sites of N acquisition). Negative effects of root herbivory on aphids could therefore arise through both reduced phloem turgor via impaired root function and lower quality phloem sap from nodule damage specifically (e.g., Murray et al., 1996). This is supported by Goldson et al. (1988b), who reported that *S. discoideus* reduced the biomass and nitrogen concentration in lucerne as a direct result of nodule damage. Indeed, there was a trend for aphids to be less able to colonize plants with weevils present, albeit not at a 95% confidence interval (*P* = 0.076). The low number of aphids at the end of this experiment may have contributed to obscuring these negative impacts. Alternatively, given that weevils began to stimulate nodulation the negative impacts of initial nodule damage on aphids may have begun to be reversed, or at least alleviated. This demonstrates the importance of considering the exact nature of root herbivory, for instance which parts of the roots are targeted by root herbivores, the sequence and duration of the attack and ultimately how these effects change over time (e.g., triggering of compensatory responses).

Root and nodule feeding also have the potential to affect neighboring plants by altering nitrogen availability in the soil (Murray and Hatch, 1994; Ayres et al., 2007). This arises because N leaks out of lacerated nodules and is taken up by co-occurring plants. Traditionally, studies between belowground and aboveground herbivores are conducted on the same shared host plant, but in this instance it could be envisaged that belowground herbivory affects aboveground herbivores on neighboring plants of different species. Essentially, root herbivory by *S. discoideus* may reduce the quality of lucerne while indirectly improving the quality of neighboring host plants. Where aphids can feed on both lucerne and the neighboring plant, this may cause aphids to migrate between plants.

Results suggest that the contrasting effects of eT and eCO_2_ on weevils likely occurred through changes in root nodulation patterns. Further study, including repeated experimental runs to reduce problems associated with pseudoreplication, should be undertaken to determine how nodule feeders belowground will affect aphids feeding aboveground, with potential pest management implications for the lucerne industry, as well as other legumes that are attacked by both insects. Incorporating such trophic complexity and multiple climatic factors represents a significant challenge for biologists, yet one that we must address to gain realistic insights into how global climate change will affect insect–plant interactions.

## Conflict of Interest Statement

The authors declare that the research was conducted in the absence of any commercial or financial relationships that could be construed as a potential conflict of interest.
